# Mutation update of *SERPING1* related to hereditary angioedema in the Chinese population

**DOI:** 10.1186/s41065-022-00242-z

**Published:** 2022-07-11

**Authors:** Xue Wang, Shubin Lei, Yingyang Xu, Shuang Liu, Yuxiang Zhi

**Affiliations:** 1 Department of Allergy & Clinical Immunology, National Clinical Research Center for Immunologic Diseases, Peking Union Medical College Hospital, Chinese Academy of Medical Sciences and Peking Union Medical College, #1 Shuaifuyuan, Wangfujing, Beijing, 100730 P.R. China; 2 Eighth-year Program of Clinical Medicine, Chinese Academy of Medical Sciences and Peking Union Medical College, Beijing, China

**Keywords:** C1 inhibitor, Hereditary angioedema, Mutational analysis, Phenotype, *SERPING1*

## Abstract

**Background:**

Hereditary angioedema (HAE) is a rare disease characterized by recurrent attacks of severe swellings of the skin and submucosa. More than 900 variants of the *SERPING1* gene associated with HAE have been identified. However, only approximately 50 variants have been identified in the Chinese population. This study aimed to update the mutational spectrum in Chinese HAE patients and provide evidence for the accurate diagnosis of HAE.

**Methods:**

A total of 97 unrelated HAE patients were enrolled in the study. Sanger sequencing and multiple ligation-dependent probe amplification analysis were used to identify the variants in the *SERPING1* gene. The variants were reviewed in a number of databases, including the Human Gene Mutation Database (HGMD) (http://www.hgmd.cf.ac.uk/) and the Leiden Open Variation Database (LOVD, https://databases.lovd.nl/shared/variants/SERPING1). The American College of Medical Genetics and Genomics-Association for Molecular Pathology (ACMG-AMP) criteria was used to determine the pathogenicity of the variants.

**Results:**

Of the 97 patients, 76 different variants were identified in 90 of them and no disease-causing variants were identified in the remaining 7 patients. Among the 76 variants, 35 variants were novel and submitted to ClinVar. Missense and in-frame variants were the most common variants (36.8%), followed by frameshift (28.9%), nonsense (14.5%), splice site (13.2%) variants, and gross deletions/duplications (6.6%).

**Conclusions:**

Our findings broaden the mutational spectrum of *SERPING1* and provide evidence for accurate diagnosis and predictive genetic counseling.

**Supplementary Information:**

The online version contains supplementary material available at 10.1186/s41065-022-00242-z.

## Background

Hereditary angioedema (HAE; OMIM #106100) is a rare, life-threatening disease characterized by unpredictable skin and submucosal swelling [1]. HAE can be caused by C1 inhibitor (C1-INH) deficiency (Type 1 HAE, HAE-1), C1-INH dysfunction (Type 2 HAE, HAE-2), or other mechanisms (HAE with normal C1-INH, HAE-nC1-INH). The genetic defect in HAE-1/2 are mutations in the *SERPING1* gene [1]. HAE-nC1-INH was first described in 2000, [2] and the pathogenic genes were gradually identified as *F12*, [3] angiopoietin-1 (*ANGPT1*), [4] plasminogen *(PLG*), [5] kininogen *(KNG1*), [6] myoferlin *(MYOF*), [7] and heparan sulfate-glucosamine 3-O-sulfotransferase 6 (*HS3ST6*) [8].

The first case of HAE in China was diagnosed by our research team in 1980, and related studies have gradually been conducted in China since then [9]. However, to date, the *SERPING1* gene is the only pathogenic gene known to be related to HAE in the Chinese population. The *SERPING1* gene encodes a highly glycosylated plasma protein, C1-INH, which irreversibly encapsulates the proteases of the kallikrein-kinin-system such as plasma kallikrein, factor XIIa, and factor XIIf in its molecule [1]. This process, termed suicide inactivation, incapacitates multiple proteases involved in the kallikrein-kinin system, complement, fibrinolysis, and coagulation pathways [1]. In HAE-1/2, the kallikrein-kinin system is overactivated and produces large amounts of bradykinin. Bradykinin mediates the active transfer of fluid into localized tissues by binding to the bradykinin B2 receptor, causing angioedema [10]. HAE-1 is caused by diverse variants of the *SERPING1* gene [11]. Missense, nonsense, frameshift, and splicing defect mutations on the *SERPING1* gene cause misfolded or truncated protein products [12]. Patients with HAE-1 are characterized by low antigenic and functional C1-INH levels [12]. However, HAE-2 occurs mainly due to missense mutations in exon 8 of the *SERPING1* gene. Such mutations affects the reactive loop and reduces the inhibitory effect of C1-INH on target proteins [1]. Patients with HAE-2 are characterized by normal or elevated antigenic but low functional C1-INH levels [13].

To date, 611 variants of the *SERPING1* gene were reported in the Human Gene Mutation Database (HGMD® Professional 2021.4, http://www.hgmd.cf.ac.uk/), and 962 variants were reported in the Leiden Open Variation Database (LOVD, https://databases.lovd.nl/shared/variants/SERPING1). Most of the variants recorded in both databases overlapped. However, in China, the HAE genotype has not been adequately studied. Our previous study reported the clinical characteristics and mutational spectrum in 48 Chinese HAE patients [14]. In these 48 patients, 25 novel mutations and 3 novel single nucleotide polymorphisms (SNPs) were identified [14]. However, no large deletions and insertions have been reported in Chinese HAE patients. Therefore, the present study aimed to provide additional information on the genotype of HAE among Chinese patients to promote the accurate diagnosis of HAE.

## Materials and methods

### Study subjects

From 2013 to 2022, 97 unrelated patients diagnosed with HAE at the Department of Allergy, Peking Union Medical College Hospital, were enrolled in the study. The diagnosis of HAE was made accroding to the US HAEA medical advisory board 2020 guidelines for the management of hereditary angioedema [15] and the international WAO/EAACI guideline for the management of hereditary angioedema [13]. In brief, HAE was diagnosed according to the typical clinical manifestation of recurrent swelling and abnormal laboratory test results. Patients with reduced C1-INH concentration and function as well as reduced C4 levels were diagnosed as having HAE-1. Patients with normal or elevated C1-INH concentration, reduced C1-INH function and reduced C4 levels were diagnosed as having HAE-2. Sixty-six unrelated healthy individuals were included as controls. All the subjects signed an informed consent form under a research protocol approved by the Research and Ethics Board of the Peking Union Medical College Hospital (Approval number: HS-2402).

### Complement measurements

Serum C1-INH antigen levels were measured using the BNTM II System (Dade Behring Marburg GmbH, Marburg, Germany). Levels of functional C1-INH were determined by using a Chromogenic Kit (Immuno Chrom, Vienna, Austria), whereas serum levels of complement 4 (C4) were determined via immunonephelometry using C4 Reagent, 29,100 Test Cartridge (Beckman Coulter, CA, USA).

### Polymerase chain reaction (PCR) amplification and sequencing analysis

Genomic DNA was isolated from EDTA-containing whole blood using the QIAamp DNA Blood Mini Kit (Qiagen GmbH, Hilden, Germany). The concentrations of DNA were detected at 260 nm and 280 nm using a laboratory spectrophotometer (Thermo Fisher Scientific, USA). Polymerase chain reaction (PCR) was used to amplify the promoter, noncoding exon 1, seven coding exons, and intron–exon boundaries of the *SERPING1* gene. Primers and PCR conditions are shown in supplemental Table S1. PCR products were sequenced using ABI 3730xl DNA Sequencer (Applied Biosystems, Foster, VA, USA) after purification. Sequence alignment comparison was performed with the reference sequence (NCBI Reference Sequence: NM_000062.2). Multiplex ligation-dependent probe amplification (MLPA) analysis was also applied. The SALSA® MLPA® Probemix P243-B1 SERPING1-F12 kit (MRC-Holland, Amsterdam, The Netherlands) was used to detect deletions or duplications of one or more exons of *SERPING1*. MLPA data were analyzed using the COFFALYSER® software (MRC-Holland). *SERPING1* variants were numbered according to the recommended CDS numbering system provided by the Human Genome Variation Society (HGVS, http://www.hgvs.org/rec.html). For amino acid positions, historical numbering based on the 478-residue mature protein was used. These variants were reviewed in public databases, including the Human Gene Mutation Database (HGMD) (http://www.hgmd.cf.ac.uk/) and the Leiden Open Variation Database (LOVD, https://databases.lovd.nl/shared/variants/SERPING1), to determine whether they have been previously reported as pathogenic.

### In silico analysis

The VarCards web server (http://159.226.67.237/sun/varcards/) was used to interpret novel missense variations. It is a comprehensive clinical and genetic database for coding variants in the human genome. This web server predicts the effect of missense mutations on proteins through 23 in silico predictive algorithms, including SIFT, Polyphen-2, and MutationTaster [16]. For intron variants, we used ESE finder 3.0 (http://krainer01.cshl.edu/cgi-bin/tools/ESE3/esefinder.cgi?process=home) to predict the effect on alternative splicing.

## Results

The average age of the 97 enrolled HAE patients was 36.0 years. Of the 97 patients, 96.9%, 69.1%, and 66.0% reported skin, gastrointestinal, and laryngeal swellings, respectively. More than 90% of patients were receiving danazol or tranexamic acid for long-term prophylaxis at the time of the study. Specifically, because the first-line long-term prophylaxes recommended by the international WAO/EAACI guidelines were not approved in China during the study period, these patients were being treated with danazol except for 2 patients younger than 16 years of age who were being treated with tranexamic acid. In addition, 8.2% of the patients had other complications, 2 patients had allergic rhinitis, 2 patients had nephritis, 1 patient had hypertension, 1 patient had hyperlipidemia, 1 patient had hepatitis B, and 1 patient had Sjögren's syndrome (Table 1).

**Table 1:** Demographic characteristics and clinical manifestations of 97 unrelated Chinese HAE patients

Clinical characteristics	Mean ± SD or n (%)
Female	56 (57.7)
Age (years)	36.0 ± 12.7
HAE-1	91 (93.8)
HAE-2	6 (6.2)
Positive family history	64 (66.0)
Onset age (years)	18.4 ± 9.4
Skin edema	94 (96.9)
Gastrointestinal edema	67 (69.1)
Laryngeal edema	64 (66.0)
Need for long-term prophylaxis	88 (90.7)
Have other complications	8 (8.2)

We identified 76 different variants in 90 unrelated HAE patients, 35 of which were novel (Table 2). None of the novel variants were detected in the 66 unrelated controls. Novel variants were submitted to ClinVar with ClinVar accession numbers from SCV001977563 to SCV001977593, SCV002043721, and SCV002522446. The pathogenicity classification was based on the ACMG-AMP variant classification criteria [17]. In addition, seven patients were not identified as carrying disease-causing variants. Their demographic characteristics and clinical presentation can be seen in Table S2. Therefore, the sensitivity of the sequencing method applied in this study was 92.8% in Chinese HAE patients.

**Table 2:** *SERPING1* mutations identified in Chinese HAE patients

Region	DNA change	Consequences	Predictive algorithm					Laboratory test		
			SIFT^a^	Polyphen-2^b^	MutationTaster^c^	Clinical classification^d^	No. of patients	C1-INH protein (g/L)^e^	C4 protein (g/L)^f^	References
Exon 2	c.1A > G	p.(Met1Val)	0.022	0.941	DC(1)	pxathogenic	1	0.04	0.015	[18]
Exon 2	c.44del	p.(Leu15Argfs*64)				likely pathogenic	1	0.04	0.01	[9]
Exon 2	c.49G > A	p.(Gly17Arg)	0.003	0.011	DC(1)	likely pathogenic	2	0.05	0.06	[19]
								0.16	0.043	
Exon 3	c.74del	p.(Asn25Metfs*54)				pathogenic	1	0.09	0.093	This study
Exon 3	c.100C > A	p.(Pro34Thr)	0.136	0.975	P(1)	VUS	1	0.13	0.053	This study
Exon 3	c.120_121del	Increased exon 3 skipping				pathogenic	1	0.04	0.015	[20]
Exon 3	c.172_181del	p.(Pro58Argfs*18)			DC(1)	pathogenic	1	0.03	0.006	This study
Exon 3	c.197dup	p.(Thr67Aspfs*15)				pathogenic	1	0.05	0.218	This study
Exon 3	c.229A > T	p.(Lys77*)			DCA(1)	pathogenic	1	0.04	0.024	This study
Exon 3	c.232del	p.(Ile78*)				pathogenic	1	0.03	0.005	This study
Exon 3	c.322C > T	p.(Gln108*)			DCA(1)	pathogenic	1	0.04	0.033	[21]
Exon 3	c.377del	p.(Pro126Leufs*22)				pathogenic	1	0.11	0.06	This study
Exon 3	c.403_404del	p.(His136Phefs*120)				pathogenic	1	0.09	0.094	[11]
Exon 3	c.508 T > C	p.(Ser170Pro)	0	1	DC(1)	pathogenic	1	0.04	0.011	[22]
Exon 3	c.509C > T	p.(Ser170Phe)	0	1	DC(1)	likely pathogenic	1	0.06	0.041	[23]
Exon 3	c.538C > T	p.(Gln180*)			DCA(1)	likely pathogenic	1	0.06	0.04	This study
Exon 3	c.550G > T	Exon 3 skipping	0	1	DC(1)	pathogenic	2	0.05	0.002	[20]
								0.08	0.076	
Exon 3	c.550G > A	Exon 3 skipping	0	1	DC(1)	pathogenic	1	0.04	0.028	[24]
Intron 3	c.550 + 1G > T	Splicing defect				pathogenic	1	0.05	0.016	This study
Intron 3	c.550 + 1G > A	Splicing defect				pathogenic	1	0.20	0.046	[25]
Exon 4	c.623dup	p.(Ala209Glyfs*48)				pathogenic	1	0.15	0.200	This study
Exon 4	c.635dup	p.(Phe213Leufs*44)				pathogenic	1	0.07	0.061	This study
Exon 4	c.666_667del	p.(Gln223Aspfs*33)				pathogenic	1	0.06	0.111	[12]
Exon 4	c.669_670del	p.(Gln223Hisfs*33)				pathogenic	1	0.05	0.003	[26]
Exon 4	c.673_675del	p.(Phe225del)				pathogenic	1	0.08	0.054	This study
Intron 4	c.685 + 1G > T	Splicing defect				pathogenic	1	0.04	0.018	[12]
Intron 4	c.686-1G > A	Splicing defect				pathogenic	2	0.04	0.030	[27]
								0.07	0.052	
Exon 5	c.708 T > G	p.(Phe236Leu)	0.007	0.999	DC(0.907)	likely pathogenic	1	0.12	0.329	This study
Exon 5	c.733_736dup	p.(Ser246Lysfs*12)				pathogenic	1	0.05	0.046	This study
Exon 5	c.744_745del	p.(Arg249Serfs*7)				pathogenic	2	0.03	0.032	[28]
								0.03	0.028	
Exon 5	c.779dup	p.(Leu261Alafs*44)				pathogenic	1	0.07	0.059	This study
Exon 5	c.785dup	p.(Asn263Glnfs*42)				pathogenic	1	0.07	0.054	This study
Exon 5	c.816_818del	p.(Asn272del)				pathogenic	1	0.07	0.025	[29]
Exon 6	c.941_942insTC	p.(Phe315Profs*7)				pathogenic	1	0.06	0.058	This study
Exon 6	c.951dup	p.(Ser318Leufs*10)				pathogenic	1	0.04	0.014	This study
Exon 6	c.983_984delinsC	p.(Lys328Thrfs*13)				pathogenic	1	0.04	0.030	This study
Exon 6	c.1019del	p.(Leu340*)				pathogenic	1	0.03	0.002	This study
Intron 6	c.1030-2A > G	Splicing defect				pathogenic	1	0.04	0.037	[30]
Exon 7	c.1051del	p.(His351Thrfs*3)				pathogenic	1	0.06	0.020	This study
Exon 7	c.1094dup	p.(His365Glnfs*4)				pathogenic	2	0.05	0.019	This study
								0.05	0.073	
Exon 7	c.1100 T > G	p.(Leu367Arg)	0.001	1	DC(1)	likely pathogenic	1	0.07	0.082	This study
Exon 7	c.1121 T > C	p.(Leu374Pro)	0.006	1	DC(1)	VUS	1	0.07	0.054	ClinVar VCV000426682.2
Exon 7	c.1157_1158del	p.(Leu386Argfs*38)				pathogenic	1	0.06	0.024	[30]
Exon 7	c.1186del	p.(Leu396*)				pathogenic	1	0.08	0.063	This study
Exon 7	c.1192C > G	p.(Leu398Val)	0.141	0.902	P(1)	likely pathogenic	1	0.09	0.101	This study
Exon 7	c.1193 T > G	p.(Leu398Arg)	0.002	1	P(0.915)	likely pathogenic	1	0.05	0.016	This study
Exon 7	c.1223A > G	p.(Asp408Gly)	0.006	0.999	DC(0.987)	pathogenic	1	0.07	0.022	[31]
Intron 7	c.1249 + 2 T > C	Splicing defect				pathogenic	1	0.04	0.034	This study
Intron 7	c.1250-2A > G	Splicing defect				pathogenic	1	0.25	0.026	[21]
Exon 8	c.1269 T > A	p.(Tyr423*)			DC(1)	pathogenic	1	0.03	0.01	This study
Exon 8	c.1289 T > C	p.(Leu430Pro)	0.001	1	DC(1)	pathogenic	1	0.08	0.071	[12]
Exon 8	c.1289 T > G	p.(Leu430Arg)	0.001	0.999	DC(0.999)	likely pathogenic	1	0.05	0.019	This study
Exon 8	c.1312del	p.(Val438Phefs*12)				pathogenic	1	0.05	0.060	[11]
Exon 8	c.1340 T > C	p.(Leu447Pro)	0.001	1	DC(1)	pathogenic	1	0.12	0.142	[26]
Exon 8	c.1342G > T	p.(Glu448*)			DC(1)	pathogenic	1	0.05	0.049	[14]
Exon 8	c.1351G > A	p.(Glu451Lys)	0.042	1	DC(1)	pathogenic	1	0.05	0.060	[32]
Exon 8	c.1356_1357del	p.(Val454Glyfs*18)				pathogenic	1	0.05	0.018	[33]
Exon 8	c.1373C > T	p.(Ala458Val)	0.004	1	P(0.960)	pathogenic	1	0.13	0.053	[34]
Exon 8	c.1379C > G	p.(Ser460Cys)	0.005	1	P(1)	likely pathogenic	1	0.14	0.044	This study
Exon 8	c.1396C > T	p.(Arg466Cys)	0.003	0.969	DC(1)	pathogenic	1	0.56	0.007	[35]
Exon 8	c.1396C > A	p.(Arg466Cys)	0.013	0.255	DC(1)	pathogenic	1	0.64	0.027	[36]
Exon 8	c.1397G > T	p.(Arg466Leu)	0.007	0.067	DC(1)	pathogenic	1	0.35	0.016	[37]
Exon 8	c.1397G > A	p.(Arg466His)	0.005	0.666	DCA(1)	pathogenic	3	0.37	0.021	[35]
								0.36	0.043	
								0.37	0.015	
Exon 8	c.1420C > T	p.(Gln474*)			DC(1)	pathogenic	1	0.04	0.008	[38]
Exon 8	c.1422G > C	p.(Gln474His)	0.2	0.996	P(0.725)	likely pathogenic	1	0.05	0.009	This study
Exon 8	c.1423C > T	p.(Gln475*)			DC(1)	pathogenic	1	0.06	0.109	[38]
Exon 8	c.1424A > C	p.(Gln475Pro)	0.006	0.998	DC(0.961)	likely pathogenic	1	0.09	0.101	This study
Exon 8	c.1425G > T	p.(Gln475His)	0.074	0.174	DC(0.865)	likely pathogenic	1	0.05	0.003	This study
Exon 8	c.1480C > T	p.(Arg494*)			DC(1)	pathogenic	7	0.05	0.050	[24]
								0.06	0.056	
								0.05	0.048	
								0.07	0.084	
								0.08	0.066	
								0.05	0.101	
								0.05	0.054	
Exon 8	c.1481G > T	p.(Arg494Leu)	0.004	0.999	DC(0.994)	pathogenic	2	0.05	0.128	[12]
								0.05	0.011	
Exon 8	c.1492C > T	p.(Pro498Ser)	0	1	DC(1)	pathogenic	1	0.05	0.031	[24]
Exon 1–2		Deletion of exon 1–2				pathogenic	1	0.04	0.031	[26]
Exon 3–4		Duplication of exon 3–4				pathogenic	1	0.05	0.100	This study
Exon 4		Deletion of exon 4				pathogenic	3	0.04	0.049	[21]
								0.07	0.045	
								0.04	0.018	
Exon 1–4		Deletion of exon 1–4				pathogenic	1	0.04	0.058	[39]
Exon 2–4		Deletion of exon 2–4				pathogenic	1	0.06	0.041	This study

^a^ SIFT® (Sorting Intolerant From Tolerant) is a program that predicts whether an amino acid substitution affects protein function. SIFT scores range from 0.0 (harmful) to 1.0 (tolerable). This score can be interpreted as follows: variants with scores in the range of 0.0 to 0.05 are considered to be harmful

^b^ PolyPhen-2® (Polymorphism Phenotyping v2) is a tool which predicts possible impact of an amino acid substitution on the structure and function of a human protein using straightforward physical and comparative considerations. The score can be interpreted as follows: 0.0 to 0.15 – Variants with scores in this range are predicted to be benign; 0.15 to 1.0 – Variants with scores in this range are possibly damaging; 0.85 to 1.0 – Variants with scores in this range are more confidently predicted to be damaging

^c^ Mutation Taster® is a free web-based application to evaluate DNA sequence variants for their disease-causing potential. In order to predict the potential pathogenicity of an alteration, each variant is distributed between Disease Causing (DC), according to NCBI ClinVar, and Polymorphism (P), according to the 1000 Genomes Project, with corresponding probability in brackets

^d^ Clinical classification is based on the ACMG-AMP criteria for variant classification supporting pathogenicity

^e^The normal range of C1-INH protein is 0.21–0.39 g/L

^f^The normal range of C4 protein is 0.100–0.400 g/L

### Missense and in-frame mutations

We identified 26 missense variants in 28 unrelated patients, 10 of which were novel (Table 2). A total of 16 previously described missense variants, including c.1A > G;p.(Met1Val), c.49G > A;p.(Gly17Arg), c.508 T > C;p.(Ser170Pro), and etc., were identified in the present study. In HAE-2, four previously reported variants were detected. These four variants include c.1396C > T;p.(Arg466Cys), c.1396C > A;p.(Arg466Cys), c.1397G > T; p.(Arg466Leu), and c.1397G > A; p.(Arg466His). All of these variants are located in exon 8 and affect Arg^466^. Arg^466^ has been identified as a critical residue of the active center loop, and variants of codon 466 are the most frequent cause of HAE-2. HAE-2 patients carrying the Arg^466^ variants had normal or elevated antigenic but low functional C1-INH levels, and low C4 levels (Table S3).

In addition, 10 novel variants c.100C > A;p.(Pro34Thr), c.708 T > G;p.(Phe236Leu), c.1100 T > G;p.(Leu367Arg), c.1192C > G;p.(Leu398Val), c.1193 T > G;p.(Leu398Arg), c.1289 T > G;p.(Leu430Arg), c.1379C > G;p.(Ser460Cys), c.1422G > C;p.(Gln474His), c.1424A > C;p.(Gln475Pro), and c.1425G > T;p.(Gln475His) were identified. Mutations c.1192C > G;p.(Leu398Val) and c.1424A > C;p.(Gln475Pro) were detected in the same family, and all three symptomatic members of this lineage carried both mutations (Figure S1). However, there was a significant clinical heterogeneity in these three patients. One patient developed the disease at age 14, had previous skin, gastrointestinal and laryngeal edema; whereas the other two patients first developed HAE at ages 25 and 23, respectively, both presented only with gastrointestinal edema. Therefore, the genetic variant was not sufficient to explain the phenotype of the patients. The cause of the biological plausibility of the difference in clinical phenotype in the 3 individuals with the same 2 variants remains to be investigated. In addition, a novel in-frame deletion c.673_675del;p.(Phe225del) was identified in one patient.

### Nonsense and frameshift mutations

A total of 11 nonsense and 22 frameshift mutations were identified in 41 unrelated patients. Among these mutations, 6 nonsense and 14 frameshift mutations were newly identified (see Table 2). For c.744_745del;p.(Arg249Serfs*7) and c.1094dup;p.(His365Glnfs*4), each of them was uniquely identified in a total of 2 patients. The previously reported c.1480C > T;p.(Arg494*) was identified in 7 patients. Other nonsense and frameshift mutations were found in different patients.

### Splicing mutations

We detected 10 splicing mutations in 12 patients, 2 of which had not been reported previously. Mutation c.686-1G > A presented in 2 unrelated patients. This mutation may affect alternative splicing of *SERPING1* through regulation of serine/arginine splicing factor (SRSF) 1 [40]. Mutation c.1030-2A > G may affect SRSF2-mediated splicing. In particular, the splicing effects of c.550G > T, c.550G > A, and c.120_121del have been demonstrated to increase exon 3 skipping and the three mutations are therefore not considered as real missense or deletion variants [20].

### Gross deletions and duplications

MLPA analysis revealed gross deletion of exon 4 in 3 patients. Exon 4 of *SERPING1* is a hotspot for large fragment deletions because *Alu* has a high repeat density in introns 3 and 4 [41]. The *Alu* sequences are movable repetitive elements which may represent hotspots for nonhomologous recombination leading to various hereditary diseases [11]. In addition, large deletion of exon 1 and 2, exon 2 – 4, and exon 1 – 4 was identified in 3 patients, respectively. Duplication of exon 3 and 4 was identified in 1 patient.

### Polymorphism

Six previously described rs28362945 (c.51 + 101G > A), rs1005510 (c.52-130C > T), rs11546660 (c.167 T > C), rs11229063 (c.685 + 88G > A), rs2511988 (c.1030-20A > G), and rs4926 (c.1438G > A) SNPs were identified in this study.

### Family study

In this study, 35 novel variants were identified. To support the pathogenicity of the novel variants, family studies were conducted. Unfortunately, only 5 patients carrying the novel variants provided blood samples from their family members. The c.172_181del, c.229A > T, c.232del, c.1051del, c.1192C > G, and c.1424A > C variants co-segregated with the disease in these five families. Of the remaining novel variants, 16 were nonsense variants or frameshift variants. These 16 novel variants create premature stop codons, which result in the termination of mRNA translation and the synthesis of truncated protein products [42]. These premature stop codons are all located upstream of the reactive loop (RCL) of C1-INH [42]. As a result, the synthesized protein will lack the RCL and could not identify the target proteases, resulting in a lack of inhibitory function of C1-INH [42]. In addition, there are also 2 novel large defects and 2 novel splicing mutations. Accroding to the ACMG-AMP criteria, nonsense, frameshift, canonical ± 1 or 2 splice sites, initiation codon, and large defects can provide very strong evidence of pathogenicity [17]. The 2 large defects mutations, the 2 splicing mutations, and the 16 nonsense or frameshift variants can be classified as pathogenic accroding to the ACMG-AMP criteria. Therefore, family studies have not been undertaken for the total 20 novel variants already classified as pathogenic. For the remaining 9 novel missense and in-frame mutations, we did not perform family studies because patient carrying the 9 novel missense and in-frame mutations refused to provide parental blood samples or were unable to provide blood samples due to the death of both parents.

## Discussion

In this study, 76 different variants were identified in 90 unrelated Chinese patients with HAE, 46.1% of which were novel. This study expands the mutational spectrum of HAE and provides evidence for accurate diagnosis and predictive genetic counseling. The sensitivity of mutational analyses applied in this study is 92.8%, which is comparable with other studies reporting a sensitivity of ≥ 82% [21, 43, 44]. We failed to find disease-causing mutations in seven patients. One possible reason is that the disease-causing mutations are located outside the sequenced region. Mutations in deep intronic region of HAE have been reported earlier. Sofia Vatsiou et al. reported the novel mutation c.-22-155G > T in intron 1 of the *SERPING1* gene, which was curated as pathogenic according to the American College of Medical Genetics and Genomics 2015 guidelines [45]. In the same year, Pavla Hujová et al. reported c.1029 + 384A > G, a novel deep intronic mutation in intron 6, is responsible for HAE. This mutation results in de novo donor splice site creation and subsequent pseudoexon inclusion [46].

**Fig. 1:**
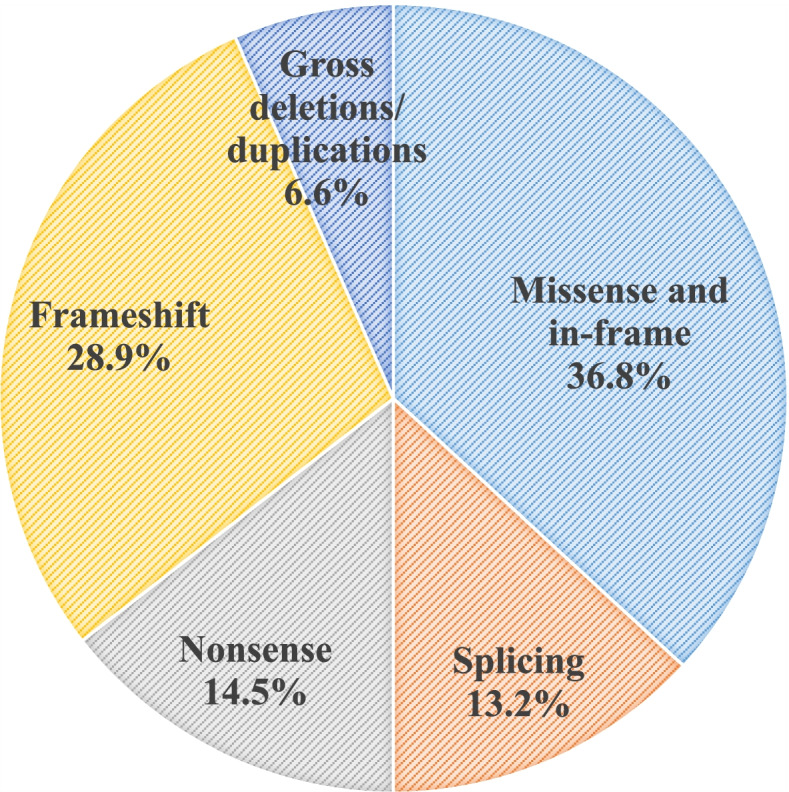
Distribution of the *SERPING1* mutation types in Chinese HAE patients

**Fig. 2:**
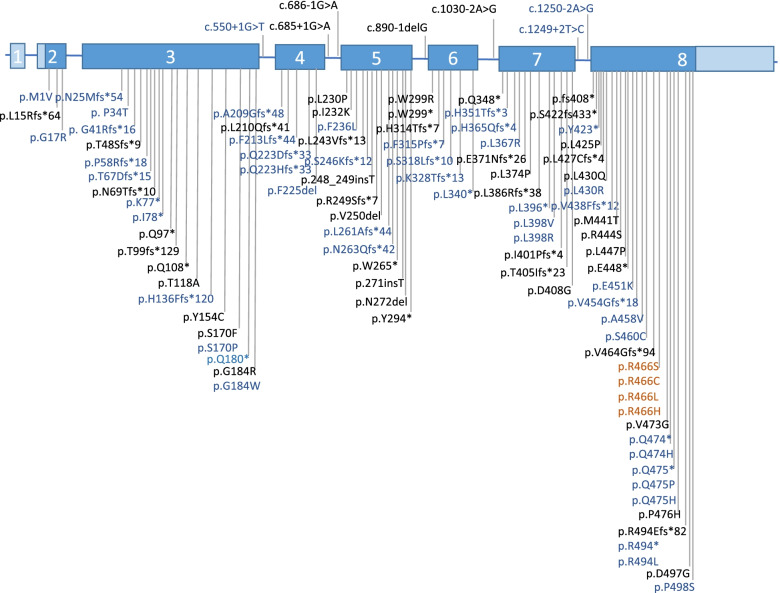
Genetic variants identified in Chinese HAE patients. Exons are numbered 1–8 and are drawn to scale in blue, with coding sequence in dark blue. Variants in blue font are reported in this study; variants in black font are reported in other studies; variants in dark red font are identified in HAE-2

The mutations that cause HAE are diverse in the Chinese population. Missense and in-frame mutations are the most common types of mutations, accounting for 36.8% of all mutations, followed by frameshift mutations (28.9%), nonsense mutations (14.5%), splice site mutations (13.2%), and gross deletions and duplications (6.6%) (Fig. 1). Figure 2 shows the disease-causing variants that have been identified in Chinese HAE patients [9, 14, 42]. Of these variants, 22 were found in exon 8, accounting for nearly one-third of all variants (Figure S2). Exon 8 appears to be a mutational hotspot in Chinese HAE patients as well as HAE patients from other countries [47]. This may be due to the fact that exon 8 contains the critical hinge region and the reaction center of the C1 inhibitor molecule [48]. Frameshift mutations and nonsense mutations account for nearly half of all mutations. These 2 mutation types result in a premature stop codon, or a nonsense codon in the transcribed mRNA [49]. When ribosomes encounter a premature stop codon, a nonsense-mediated mRNA decay is triggered, which results in a truncated, incomplete, and usually nonfunctional protein product [49]. Our previous study also showed that frameshift mutations and nonsense mutations led to a reduced expression of *SERPING1* mRNA in peripheral blood [42].

In the Chinese HAE cohort, patients with HAE-1 and HAE-2, respectively, accounted for 93.8% and 6.2% of all HAE patients. The increased proportion of HAE-2 compared with our previously reported 1.27% may be a more comprehensive response to the characteristics of Chinese HAE patients after expanding the sample size [9, 50]. The updated proportion of HAE-2 in China is comparable to that found in Denmark, Spain, and Germany [43, 44, 51]. Besides, the percentage of gastrointestinal edema in Chinese HAE patients was 69.1%, which was higher compared with our previous report of 34.2% [9, 50]. The percentage reported in the current study is higher than that in Japan (45.0%) [52] and Taiwan, China (18.2%), [53] but lower than those in Western countries (German: 97.0% and Danish: 96.1%) [51, 54]. Moreover, the higher percentage of gastrointestinal edema reported in this study indicates that HAE is gradually being recognized in China, and more patients with gastrointestinal edema are being diagnosed. In addition to gastrointestinal edema, the percentage of laryngeal edema in Chinese HAE patients in the current study (66.0%) was also slightly higher compared with that of our previous report (58.9%) [50].

Several limitations should be noticed. First, this is a single center analysis although the patients are from all over China. Second, we did not find HAE patients with normal C1-INH levels. This does not mean that the *SERPING1* gene is the only pathogenic gene in Chinese HAE patients. Given the vast population of China, it is possible that there are HAE patients with normal C1-INH levels that have not been identified because of the absence of commercially available biomarkers. Third, we failed to find disease-causing mutations in seven HAE patients. The pathogenic mutations in these patients may be located outside of the sequenced region. The intronic region of *SERPING1* will be sequenced in a subsequent study. Fourth, although we reported 35 novel mutations, functional validation was not performed, which can be explored in future studies.

## Conclusions

HAE is a rare disease with great heterogeneity in both genotype and clinical phenotype. This study updated the clinical phenotypic characteristics and mutational spectrum of Chinese HAE patients. 41 previously reported variants and 35 novel variants were identified in 90 unrelated patients. This study provides more comprehensive information on the genetic characteristics and clinical presentation of Chinese HAE patients.

## Supplementary Information

**Additional file 1:** **Table S1.** Primers and PCR conditions. **Table S2.** Demographic characteristics and clinical manifestations of 7 patients without genetic variants. **Table S3.** The antigenic and functional C1-INH levels and C4 levels corresponding to the genetic variants of HAE-2. **Figure S1. **Mutations c.1192C>G;p.(Leu398Val) and c.1424A>C;p.(Gln475Pro) were detected in all three symptomatic members of this lineage. **Figure S2.** Proportional distribution of variants on the *SERPING1* gene.

## Data Availability

The datasets used and/or analysed during the current study are available from the corresponding author on reasonable request.
