# Supplementary material for: Mutation update of SERPING1 related to hereditary angioedema in the Chinese population
Source: Hereditas. 2022 Jul 11;159:28. doi: 10.1186/s41065-022-00242-z (PMC9277798; doi:10.1186/s41065-022-00242-z)
Supplement: Supplementary file 1 — Additional file 1: Table S1. Primers and PCR conditions. Table S2. Demographic characteristics and clinical manifestations of 7 patients without genetic variants. Table S3. The antigenic and functional C1-INH levels and C4 levels corresponding to the genetic variants of HAE-2. Figure S1. Mutations c.1192C>G;p.(Leu398Val) and c.1424A>C;p.(Gln475Pro) were detected in all three symptomatic members of this lineage. Figure S2. Proportional distribution of variants on the SERPING1 gene. [file 41065_2022_242_MOESM1_ESM.docx]

**Table S1.** Primers and PCR conditions

|  | Primers | Product | Annealing temperature (℃) |
| --- | --- | --- | --- |
| E1-F | CCCACCTACCAGGGGATTT | 408 | 58 |
| E1-R | GGGGTAGGAGGAGGCAGAA |  |  |
| E2-F | TTGGGGAAAACAAAACAGAGG | 518 | 60 |
| E2-R | GGAGGAGTAGGCTGAGAAAAGTG |  |  |
| E3-F | CCACCCTCACCCTGTATTGC | 900 | 58 |
| E3-R | GCAATCGTGCCTATTACATCACT |  |  |
| E4-F | CTGGTCCCCAACCCTCAT | 521 | 58 |
| E4-R | GCCTGGCTTTCTGCTTCTT |  |  |
| E5-F | TCTGGGTTTACCTTCTTTGGG | 453 | 58 |
| E5-R | GAAGCCAGGAGGGGAAGAA |  |  |
| E6-F | GTCCTTCTTCCCCTCCTGG | 445 | 58 |
| E6-R | AGGAGAAAAGATAGGGTGGAAAT |  |  |
| E7-F | CTTAGGTCTGACTGATGCTTGTTG | 609 | 58 |
| E7-R | TGGGTCGGTGTTCTGGTTT |  |  |
| E8-F | TCAGGACAAAGGTCTCCATCA | 744 | 60 |
| E8-R | TGAGATGGGAGGATTGTTTGA |  |  |

**Table S2.** Demographic characteristics and clinical manifestations of 7 patients without genetic variants

| Patient | Sex | Age | Family history | Onset age | Skin edema | Gastrointestinal edema | Laryngeal edema | C1-INH protein (g/L)* | C4 protein (g/L)* |
| --- | --- | --- | --- | --- | --- | --- | --- | --- | --- |
| 1 | F | 41 | yes | 16 | yes | yes | yes | 0.09 | 0.09 |
| 2 | F | 51 | yes | 50 | yes | yes | no | 0.11 | 0.112 |
| 3 | F | 70 | yes | 15 | yes | yes | yes | 0.04 | 0.05 |
| 4 | M | 18 | yes | 8 | yes | yes | yes | 0.03 | 0.036 |
| 5 | F | 64 | yes | 20 | yes | yes | yes | 0.05 | 0.04 |
| 6 | M | 28 | no | 23 | yes | yes | yes | 0.12 | 0.112 |
| 7 | M | 41 | no | 20 | yes | yes | yes | 0.06 | 0.065 |

*The normal range of C1-INH protein is 0.21-0.39 g/L; The normal range of C4 protein is 0.100-0.400 g/L.

**Table S3.** The antigenic and functional C1-INH levels and C4 levels corresponding to the genetic variants of HAE-2

| Patient | Variant | C1-INH protein (g/L) | C4 protein (g/L) | C1-INH function % |
| --- | --- | --- | --- | --- |
| 1 | c.1396C>T;p.(Arg466Cys) | 0.56 | 0.007 | 22 |
| 2 | c.1396C>A;p.(Arg466Cys) | 0.64 | 0.027 | 32 |
| 3 | c.1397G>T;p.(Arg466Leu) | 0.35 | 0.016 | 37 |
| 4 | c.1397G>A;p.(Arg466His) | 0.37 | 0.021 | 19 |
| 5 | c.1397G>A;p.(Arg466His) | 0.36 | 0.043 | 20 |
| 6 | c.1397G>A;p.(Arg466His) | 0.37 | 0.015 | 28 |

*The normal range of C1-INH protein is 0.21-0.39 g/L; The normal range of C4 protein is 0.100-0.400 g/L; The normal range of C1-INH function is 70%-130%

**Figure S1.** Mutations c.1192C>G;p.(Leu398Val) and c.1424A>C;p.(Gln475Pro) were detected in all three symptomatic members of this lineage

**Figure S2.** Proportional distribution of variants on the *SERPING1* gene
